# Precise estimation of soil organic carbon stocks in the northeast Tibetan Plateau

**DOI:** 10.1038/srep21842

**Published:** 2016-02-24

**Authors:** Ren-Min Yang, Gan-Lin Zhang, Fei Yang, Jun-Jun Zhi, Fan Yang, Feng Liu, Yu-Guo Zhao, De-Cheng Li

**Affiliations:** 1 State Key Laboratory of Soil and Sustainable Agriculture, Institute of Soil Science, Chinese Academy of Sciences, Nanjing 210008, China; 2 University of the Chinese Academy of Sciences, Beijing 100049, China

## Abstract

There is a need for accurate estimate of soil organic carbon (SOC) stocks for understanding the role of alpine soils in the global carbon cycle. We tested a method for mapping digitally the continuous distribution of the SOC stock in three dimensions in the northeast of the Tibetan Plateau. The approach integrated the spatial distribution of the mattic epipedon which is a special surface horizon widespread and rich in organic matter in Tibetan grasslands. Prediction models resulted in high prediction accuracy. An average SOC stock in the mattic epipedon was estimated to be 4.99 kg m^−2^ in a mean depth of 14 cm. The amounts of SOC in the mattic epipedon, the upper 30 cm and 50 cm accounted for about 21%, 80% and 89%, respectively, of the total SOC stock in the upper 1 m depth. Compared with previous estimates, our approach resulted in more reliable predictions. The mattic epipedon was proven to be an important factor for modelling the realistic distribution of the SOC stock in Tibetan grasslands. Vegetation-related covariates have the most important influence on the distribution of the mattic epipedon and the SOC stock in the alpine grassland soils of northeast Tibetan Plateau.

The soil organic carbon (SOC) pool is one of the most important reservoirs in the global C cycle^1^. This reservoir contains more C than that of the atmospheric pool and the biotic pool^2^,^3^. The role of the SOC pool is considered as a potential sink of greenhouse gases^4^,^5^,^6^. As frigid alpine area is believed more vulnerable to warming climate, to understand how SOC is stored in such an environment is critical for evaluating the feedbacks of SOC to global environmental change^7^. Therefore, accurate estimation of total SOC stocks is important for accessing the C sink capacity of soils and the change rate of SOC^8^. In addition, for application purposes, spatially explicit information of SOC in three dimensions plays a crucial role in many simulation models^9^,^10^,^11^.

The conventional approach for predicting the spatial distribution of SOC stocks is carried out by calculating the mean SOC measurements within each map unit by soil type or land use^12^,^13^,^14^,^15^. Nevertheless, this method may lead to a less reliable estimation due to the great spatial heterogeneity within each map unit and the errors of assigned average SOC values from few SOC data^16^,^17^. To overcome this problem, digital soil mapping (DSM) is deemed as an appropriate and useful technique to produce detailed information of SOC stocks from auxiliary environmental covariates^18^. The majority of recent DSM studies on SOC stocks prediction focused on a fixed soil depth, e.g. top 0.1, 0.2, 0.3 or 1.0 m^19^. Information of SOC on fixed soil depth is missing knowledge on the depth distribution that, however, is quite important for understanding the role of SOC in the global C cycle and for quantifying the environmental controls on SOC distribution^20^. Thereby, continuous distribution of SOC in vertical dimension is also necessary to investigate storage and controls of SOC. Recently, soil depth functions in combined with DSM have been used to derive three-dimensional continuously distribution of SOC^20^,^21^,^22^,^23^,^24^. Soil depth functions such as exponential depth functions or equal-area splines used in these studies are clearly advantageous for mapping SOC stocks at specific depths^22^.

In the Tibetan Plateau, alpine grassland is the most widespread ecosystem and plays an important role in the storage of SOC^25^,^26^. Specially, soils usually have intensive roots mixed topsoils in the alpine meadow areas^27^. This roots-felty topmost layer is named as “the mattic epipedon” in Chinese Soil Taxonomy^28^, which contains high SOC content^29^,^30^. Consequently, it usually leads to a sharp decrease in SOC contents with depth^25^. The estimations of SOC stocks in the Tibetan Plateau have received considerable attention in several studies^25^,^31^,^32^. However, few studies have attempted to completely address both of lateral and vertical distribution of SOC due to low sampling density and large spatial variation of SOC^25^,^32^,^33^. Furthermore, no studies have been conducted to quantify continuous depth distribution of SOC stocks in the Tibetan Plateau. In order to produce spatially explicit distribution of SOC stocks in three dimensions in this region, a combined prediction model of DSM and soil depth functions may be applicable based on easily available environmental covariates and a small number of soil samples.

In the present study, we mapped SOC stocks within the upper 1 m depth in the northeast Tibetan Plateau (Fig. 1) using soil depth functions and random forest. The specific objectives are: (1) constructing soil depth functions to describe vertical distribution of SOC with integration of the occurrence of the mattic epipedon; (2) deriving predictive models to map the soil depth functions across the study area; (3) evaluating importance of environmental covariates in controlling the spatial patterns of SOC; and (4) producing SOC maps in three dimensions by applying defined prediction models.

## Results

### Soil organic carbon at surface horizons

Summary statistics of soil organic carbon contents and stocks at surface layers are shown in Table 1. The higher SOC contents were observed in the mattic epipedon, around a mean of 38.06 kg m^−3^. Within the ordinary surface horizon, the mean value of SOC contents was 22.15 kg m^−3^. The mean SOC stock in the mattic epipedon was 5.90 kg m^−2^ to an average depth of 15 cm, while it was 2.91 kg m^−2^ to an average depth of 12 cm in the ordinary horizon. The variances of SOC contents and stocks in the mattic epipedon were lower than that in the ordinary horizon.

### Modelling SOC depth distribution

The soil depth function (Eq. 3) was applied to model the SOC vertical distribution. In total four parameters are required to fit the depth function. This function was constructed using the mattic epipedon assigned exponential decay function. For instance, the SOC content in a profile covered by the mattic epipedon was fitted by applying the defined depth function (Fig. 2). The soil in the site was Matti-Gelic Cambosols according to Chinese Soil Taxonomy^34^. This site is exhibiting typical mattic features to the depth of 18 cm. At the topmost mattic covered layer, the SOC content was 41.56 kg m^−3^ (Fig. 2). With depth, the SOC content decreased sharply to 7.03 kg m^−3^ at the depth of 90–100 cm. We applied the defined depth function to all calibration sites. The results showed that the soil depth function provided a mean *R*^*2*^ of 0.91 and a SD of 0.09 between the observed and fitted SOC content.

### Spatial prediction of soil depth functions

The parameters of the soil depth function were predicted by random forest (RF) models. Descriptive statistics of the performances of prediction models were shown in Table 2. OOB error rate was about 0.17 for the mattic epipedon classification. The mean values of OOB MSE were 0.04, 0.35 and 0.67 for log(mattic depth, m), log(Ca, kg m^−3^) and log(k), respectively.

### Importance of environmental covariates

Covariate importance revealed different environmental dominances influencing the mattic epipedon and SOC (Fig. 3). Generally, spatial coordinates (latitude and longitude) are important covariates in explaining the spatial variability of the mattic epipedon and SOC. Importance of topography, climate and vegetation covariates differs largely in RF models.

Distribution of the mattic epipedon was largely influenced by vegetation and climate covariates, such as mean annual precipitation (MAP), the normalized difference vegetation index (NDVI), mean annual temperature (MAT) and Landsat TM Band5 (B5) (Fig. 3). Although elevation shows relative large importance, the remaining topography covariates are of little contribution for the mattic epipedon prediction. For mattic depth, NDVI is the most important factor in explaining the depth of the mattic epipedon. Other vegetation covariates are also relevant for mattic depth prediction. In addition, the importance of slope length (SL), elevation, MAT and MAP are highly related to mattic depth.

Regarding the processes of SOC accumulation in the topsoil, B3 and NDVI are the most important covariates determining Ca by RF, followed by elevation, Lat, MAP, MAT, aspect and other covariates (Fig. 3). The distribution of SOC with depth (the parameter k) is mainly influenced by elevation, MAT, valley depth (VD), aspect and MAP.

### Spatial prediction and validation of SOC stocks

The capability of prediction models was evaluated on the training and independent samples. Statistics of validation indices were summarized in Table 3. The models result in reasonable predicts, with an Lin’s concordance correlation coefficient (LCCC) of 0.90 (0.89–0.90) and 0.54 (0.53–0.55), respectively, for internal and independent validations. The root mean square error (RMSE) values of independent validation ranged from 0.92 to 0.95 kg m^−2^, with a mean of 0.94 kg m^−2^, which were higher than those of internal validation. Mean error (ME) values of two validation methods are −0.06 and −0.59 kg m^−2^, suggesting that the predictions for SOC stocks are slightly negative biased.

By applying the predicted parameters of the soil depth function, we mapped the spatial distribution of SOC stocks across the study area Fig. 4, Fig. 5 and Fig. 6. Overall, the soils of the southeastern part have relatively higher SOC stocks than the rest of the area (Fig. 5). The area of the mattic epipedon was estimated to 8815 km^2^ distributed mainly in the south part of the area (Fig. 4). Fig. 6 shows the vertical distribution of SOC at the top 1 m depth along longitude 99.5°E. As expected, SOC content dominantly concentrated in the topsoils, and decreased with depth. Sites covered with the mattic epipedon have higher SOC content than other sites. In mountains areas, SOC distributions show sharp discontinuities.

The mean value of mapped SOC stock was 4.99 kg m^−2^ for the mattic epipedon to an average depth of 14 cm and a SD of 1 cm (Table 4). The average stock for the 0–30 cm layer was 5.54 kg m^−2^, that of 6.11 kg m^−2^ for the 0–50 cm layer and 6.89 kg m^−2^ for the 0–100 cm layer. Total SOC stocks were calculated based on the maps of SOC stocks, and summarized in Table 4. In the mattic epipedon, SOC was estimated at 43.95 Tg, about 21% of the total SOC of 209.87 Tg for the top 1 m soils. In the area, 168.89 Tg about 80% of the total SOC in the top 1 m depth was stored in the layer of 0–30 cm.

## Discussion

In this study, a combined model of the soil depth function and random forest was used to predict SOC distributions in three dimensions in the Tibetan grasslands. This is different with previous studies^13^,^15^,^25^ in which SOC stocks were estimated on fixed soil depth in the Tibetan Plateau. These studies were irrespective of continuous variation of SOC with depth. In addition, the use of the soil depth function makes the approach flexibly to be solved by predicting the parameters of the function using easily available environmental covariates. This is more suitable than data-driven methods in Tibetan regions, because it is difficult to conduct high density sampling at a regional scale in such areas. Furthermore, the methodology presented here addressed the mattic epipedon in modelling the SOC depth distribution, which is in contrast with the method based on a simple decay function^8^,^21^. Integrating the mattic epipedon for constructing the soil depth function was expected to result in a more realistic depth distribution of SOC. This topsoil horizon is a common pedogenetic feature in the Tibetan grasslands, which is characterized by the rich in organic matter^27^,^29^,^30^. By investigating the mattic epipedon, we were able to quantify the role of such an uppermost soil layer in storing SOC. Therefore, information on this layer is valuable for three-dimensional mapping of SOC stocks in the Tibetan grasslands.

Indices of validation provided high LCCC, and low ME and RMSE (Table 3). Although the internal accuracy determined by comparing the estimates at the calibration sites indicated promising results of the prediction models, this validation method usually overestimates the real prediction quality^35^. They suggested that validation with an independent dataset not used in calibration would be preferably for evaluating the quality of predictions. Thus, we validated predictions of SOC stocks using three independent pedons intensively sampled at a 5-cm interval. The independent validation showed that our methodology was able to provide acceptable estimates of SOC stocks with a mean LCCC of 0.54 and RMSE of 0.94 kg m^−2^ (Table 3). These results are comparable with the values reported from previous studies on three-dimensional mapping of SOC stocks, such as Mishra *et al.*^8^ (*R*: 0.34–0.75 and RMSE: 2.57–3.93 kg m^−2^), Minasny *et al.*^21^ (*R*^*2*^: 0.26–0.36 and RMSE: 0.56–2.80 kg m^−2^), Malone *et al.*^22^ (*R*^*2*^: 0.20–0.27), Kempen *et al.*^23^ (*R*^*2*^: 0.09–0.75) and Poggio and Gimona^36^ (*R*^*2*^: 0.60). In the Tibetan Plateau, Yang *et al.*^25^ predicted spatial distribution of SOC stocks using a satellite-based enhanced vegetation index at the layers of 0–30, 0–50 and 0–100 cm across entire Tibetan grasslands. Explained variances were 50%−66% for SOC stocks in their prediction models. Our results indicated that the application of the soil depth function and DSM approach would be a good choice to mapping soil carbon stocks in the Tibetan Plateau. Additionally, we examined the spatial correlation structure of the residuals and did not find any spatial structure. Therefore, prediction accuracy cannot be further improved by the regression kriging approach as implemented in studies of Odeh *et al.*^37^ and Hengl *et al.*^38^. For accuracy consideration, independent samples are deemed necessary for assessment of the model performance. The independent validation presented was based on three pedons only. Although the number of validation dataset (three pedons) was too small compared to calibration dataset (ninety-six pedons), three independent pedons were intensively sampled at a 5-cm interval. A total of sixty samples for independent validation provided reliable assessment of the model performance to some extent. To avoid biased evaluation, the validation procedure can further be improved by adding more samples from various sites.

Mattic epipedons have relatively high SOC contents and stocks compared with ordinary topmost layers (Table 1). Vertically, SOC stock in the mattic epipedon (a mean depth of 14 cm) accounted for about 21% of that in the upper 1 m (Table 4). It indicates that large amount of SOC is stored in the mattic epipedon that captures only one fourth areas in the study region. Our prediction models showed that the most important predictor is vegetation-related variable. This is consistent with the basic phenomenon of grass-roots twined in the mattic epipedon (according to Kaiser *et al.*^27^). In addition, the spatial distribution of the mattic epipedon is primarily associated with the distribution of vegetation cover. The growth of grasslands is therefore a direct factor influencing the formation process of the mattic epipedon. The influence of climate variables and elevation was also highlighted in RF models. Yang *et al.*^30^ found that the mattic epipedon only occurred in high altitude and cold regions. In such environments, permafrost conditions lead to slow decomposition rates of organic matter in topsoils^39^. Water saturation in topsoils during the rainy season was an additionally factor reducing the decomposition rates of organic matter^27^,^40^. Both factors determined the accumulation of organic matter in the mattic epipedon.

Latitude and longitude are important predictors identified by RF. The importance of geographic position could be related to the fact that environment variables, such as climate, vegetation and topography, are associated with spatial coordinates in the area. Remote-sensed images and the derived NDVI show large importance for the parameter Ca in the exponential decay function. These predictors were used to present the spatial variability of vegetation cover, biomass and productivity^41^,^42^. For SOC, vegetation is the dominant source, which determines the quantity of organic matter input in soils^7^,^43^. In the Tibetan grasslands, Yang *et al.*^25^ reported that the remote-sensed vegetation index is valuable and applicable in mapping the spatial distribution of SOC. They indicated that SOC was almost determined by plant production in such ecosystems. Moreover, climate effects on SOC distribution are also important according to our results. Previous studies found that soil moisture has the most significant influence on SOC in the Tibetan Plateau^25^,^29^,^32^. The effect of soil moisture on SOC is determined by permafrost in alpine ecosystems^29^. Due to the sensitivity of this region to climate change, permafrost degradation has been enhanced over the past decades and that further lead to soil moisture-temperature regimes^44^. A study by Liu *et al.*^32^ reported that soil moisture has a significant correlation with MAT in the northeast Tibetan Plateau. Elevation and aspect show strong impact on SOC. Elevation is highly influential in regional temperature and precipitation features in this study. Aspect usually influences microclimate. Consequently, the effect of topography variables on SOC may be mediated by vegetation and climate variables.

We applied soil depth functions to estimate SOC stocks across the study area, which results in continuous distributions of SOC stocks in three dimensions. The maps reflect the detailed information of SOC stocks and contents, with a 90-m resolution in lateral and a 1-cm resolution in vertical. The estimate of SOC stocks from this study was comparable to previous studies in the Tibetan Plateau. Yang *et al.*^25^ built multiple regression models using a satellite-based enhanced vegetation index as a predictor to map SOC stocks across the Tibetan Plateau. They reported that the average SOC stock was estimated at 4.42, 5.43 and 6.52 kg m^−2^ at the layers of 0–30, 0–50 and 0–100 cm. These estimates are lower than our predictions. The proportion of SOC in the upper 30 cm is about 68% of total SOC in the upper 1 m in their study which is lower than that of 80% in our study. These differences may be partly explained by the specific modelled SOC stocks in the mattic epipedon in this study. This comparison further indicates the importance of the mattic epipedon in SOC stocks prediction in the Tibetan Plateau. Liu *et al.*^32^ found that the average SOC stock was 7.72 kg m^−2^ at the upper 1 m depth by using statistical analysis from 14 sites in the upstream regions of the Shule River Basin in the northeast Tibetan Plateau. The real SOC stocks may be overestimated from statistics of sparse samples due to the large spatial variation of SOC in the Tibetan Plateau. Thus, the estimate from the pixel-based approach would be more reliable than sample-based statistics. It indicates that the three-dimensional mapping approach constructed in this study is promising to produce maps of SOC stocks in the Tibetan Plateau.

## Methods

### Soil samples and analysis

**Figure 1:**
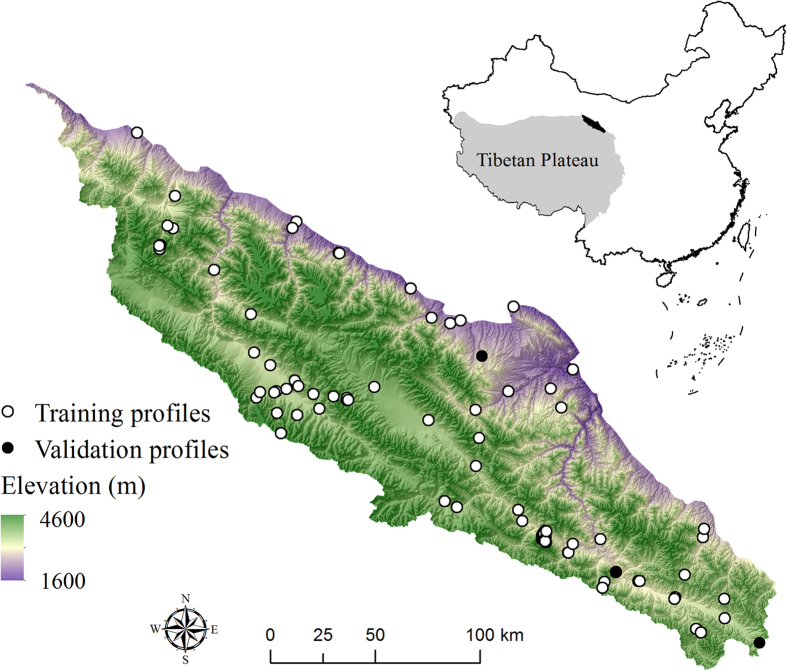
Location map of study area and 99 soil profile sites. Background is a digital elevation model (overlaid hillshading). The figure was generated by using ArcMap 10.0 (http://www.esri.com/).

**Figure 2:**
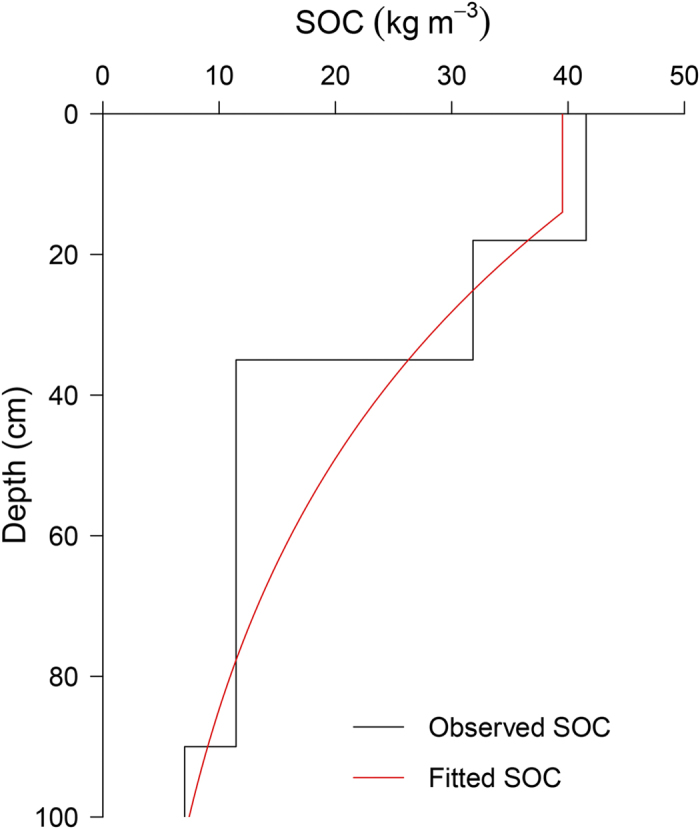
An example of applying a soil depth function on a soil profile with the mattic epipedon. Matti-Gelic Cambosols (Chinese Soil Taxonomy); 38.27°N, 99.88°E; 3009 asl, north-facing slope; MAT −0.2 °C, MAP 301 mm; Alpine meadow, NDVI 0.57.

**Figure 3:**
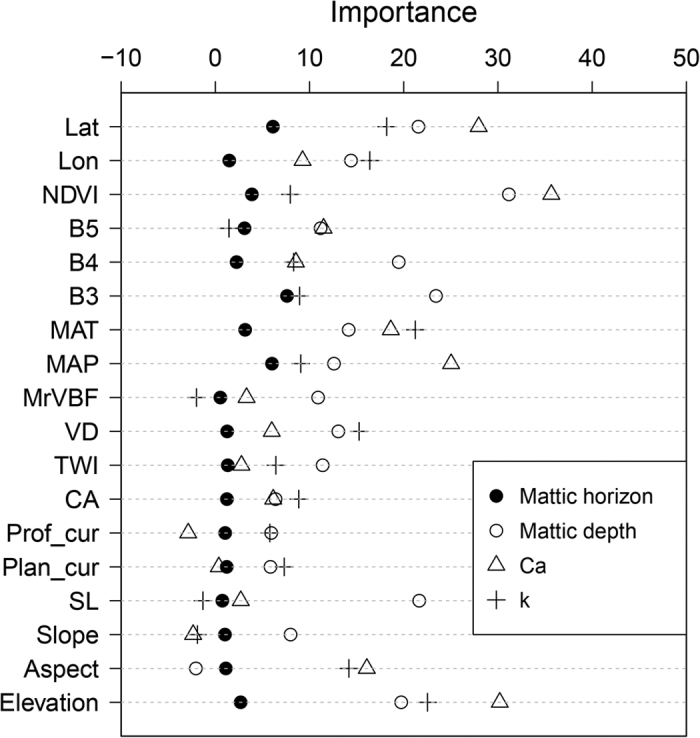
Covariate importance. Lat, latitude; Lon, longitude; NDVI, the normalized difference vegetation index; B3, Landsat TM band 3; B4, Landsat TM band 4; B5, Landsat TM band 5; MAP, mean annual precipitation; MAT, mean annual temperature; SL, slope length; Plan_cur, plan curvature; Prof_cur, profile curvature; CA, catchment area; TWI, SAGA wetness index, MrVBF, multi-resolution index of valley bottom flatness and VD, valley depth.

**Figure 4:**
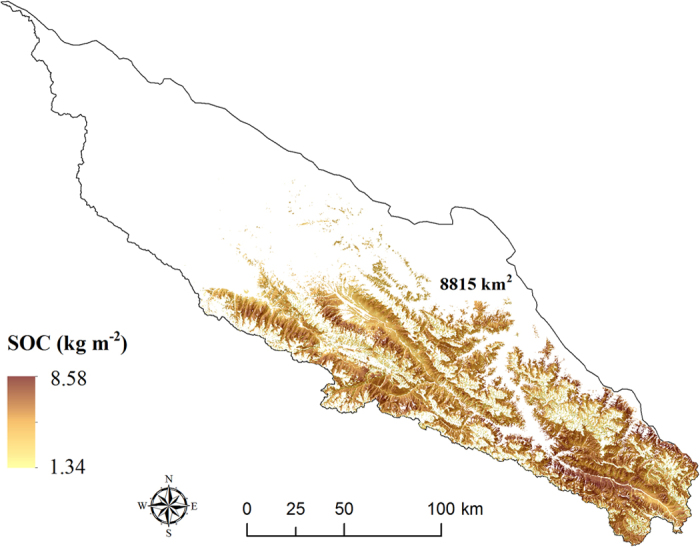
Spatial distribution of soil organic carbon stocks in the mattic epipedon (overlaid hillshading). The figure was generated by using ArcMap 10.0 (http://www.esri.com/).

**Figure 5:**
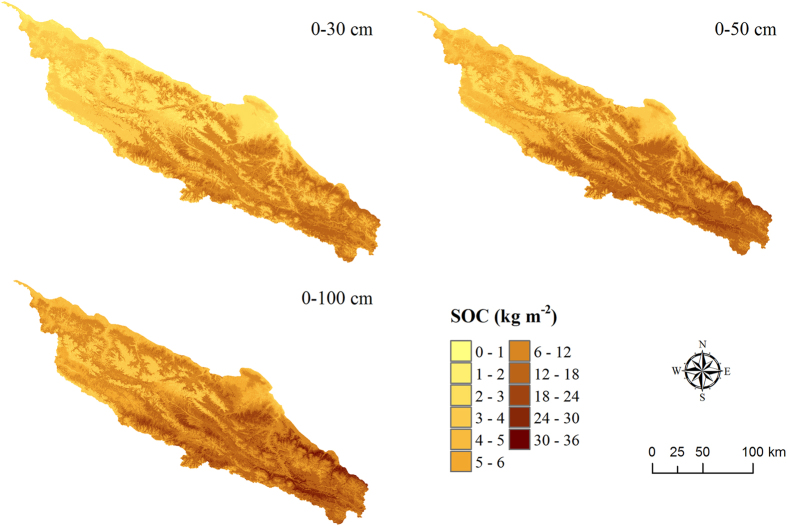
Spatial distribution of soil organic carbon stocks at 0–30, 0–50 and 0–100 cm. The figure was generated by using ArcMap 10.0 (http://www.esri.com/).

**Figure 6:**
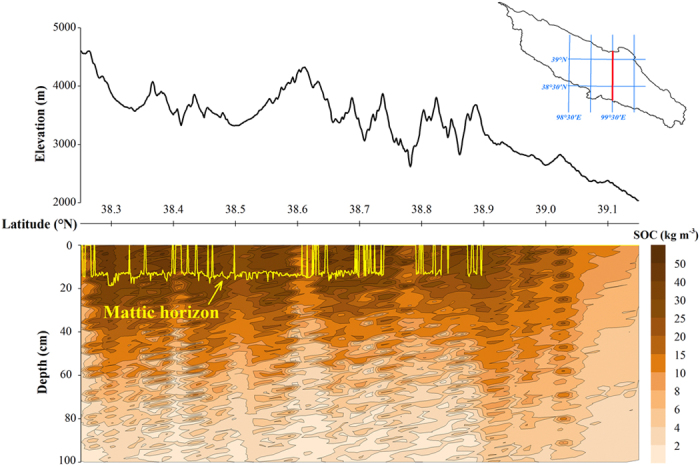
Vertical distribution of soil organic carbon content at the top 1 m depth along longitude 99.5°E.

The study area is located in the northeast margin of the Tibetan Plateau, China (Fig. 1). It covers approximately 30000 km^2^ between latitudes 37.71° and 40.03°N and longitudes 96.78° and 101.2°E. Elevation in the area is ranging from 1600 to 4600 m above sea level. This region belongs to the part of the Tibetan grasslands ecosystem, with cold and dry climate. Rainfall almost occurs between June and August. The vegetation is dominantly by alpine meadow and alpine steppe. The typical land use is grazing lands.

In the Tibetan Plateau, field sampling is difficulty due to the constraints of accessibility. In order to improve sampling efficiency, we designed a purposive sampling strategy to identify sample sites. This approach can result in a minimum number of typical soil samples to represent the variability of soil forming factors, such as topography, climate, land use and parent material. Detailed information about the method can be found in the study of Zhu *et al.*^45^. In addition, the accessibility of each designed sample site was evaluated based on traffic data. Finally, a total of ninety-nine (99) soil profiles were selected for SOC mapping (Fig. 1). Soil samples were collected during June to August in the years of 2012 and 2013. All profiles were described to a depth of 1.2 m or shallower bedrock. Ninety-six pedons were sampled from genetic horizons according to the Chinese Soil Taxonomy guidelines^34^. These samples were used for model calibration. The remaining three pedons were intensively sampled at a 5-cm interval for independent validation.

To determine SOC content, the Walkley-Black wet combustion method was used^46^. Gravel content (G) was determined by the volume percentage of the rock fragments >2 mm. Soil bulk density (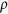, g cm^−3^) was measured from the over-dry sample at 105 °C for 12 h. The unavailable measurements of soil bulk density were calculated from SOC content by using a pedo-transfer function (PTF) (Eq. 1):


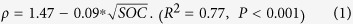


Finally, the SOC content in mass basis (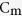, g kg^−1^) was converted to volume basis (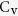, kg m^−3^) (Eq. 2):


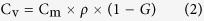


The mattic epipedon is a unique diagnostic surface horizon in Chinese Soil Taxonomy, defined as “A mat-like organic epipedon which is a complicate mixture of plant roots, including living and dead, and organic soil materials with high content of organic carbon, under the vegetation of alpine or subalpine meadow”^34^. In the Chinese Soil Taxonomy guidelines, some criterions were given for determining the occurrence of the mattic epipedon, such as thickness (>5 cm), textile roots (>50%), ratio of carbon to nitrogen (14–20), soil color, saturation (<1 month), soil bulk density (0.5–1.1 Mg m^−3^) and soil temperature (cyric). However, these properties were difficult to be measured or quantified in field. Thus, we first determined the occurrence and depth of the mattic epipedon by expert judgment, and then inspected field descriptions using measured soil properties. In the total of 99 sites, 41 pedons were found with the mattic epipedon.

### Pre-processing on environmental covariates

For digital mapping of SOC stocks, the important environmental covariates were selected from the scorpan factors defined by McBratney *et al.*^18^, including topography, climate, vegetation and spatial position. These covariates were resampled to a common grid of 90-m resolution.

A digital elevation model (DEM) was obtained from the Shuttle Radar Topography Mission terrain (STRM, 2009), with a 90-m resolution. Based on this DEM dataset, a set of ten first and second terrain attributes were calculated: elevation, aspect, slope, slope length (SL), plan curvature (Plan_cur), profile curvature (Prof_cur), catchment area (CA), SAGA wetness index (TWI), multi-resolution index of valley bottom flatness (MrVBF) and valley depth (VD). Aspect was expressed as absolute 0 to 180° to represent face from north to south. Climate data, 1 km-resolution mean annual temperature (MAT) and mean annual precipitation (MAP), were obtained from Chinese Academy of Agricultural Sciences. Landsat 5 TM imagery and the derived normalized difference vegetation index (NDVI) were used to present the spatial variability of vegetation. The remotely-sensed imagery was acquired from the Environmental and Ecological Science Data Center for West China. 21 relief-corrected images from July to September (growing season) in 2010 were mosaicked and trimmed to cover the entire study area. The vegetation predictors contain Landsat TM visible red Band 3 (B3, 0.63–0.69 μm), near infrared Band 4 (B4, 0.76–0.96 μm), short-wave infrared Band 5 (B5, 1.55–1.75 μm) and NDVI calculated by (B4 − B3)/(B4 + B3).

In addition, we produced latitude and longitude maps in a 90-m resolution as spatial position predictors. McBratney *et al.*^18^ highlighted the importance of spatial position data in mapping soil properties and concluded it as a factor in the scorpan model for describing the relationships between soil and environmental variables. They indicated that spatial coordinates have the potential to reflect some environmental variables and to explain parts of the variation of soil properties in large extent.

### Three-dimensional modelling and validation

This section describes the methodology used for three-dimensional mapping of SOC stocks. First, a step-wise exponential depth function was used to fit SOC depth distribution at calibration sites. In this step, the mattic epipedon was highlighted for obtaining a realistic estimate. Second, a combined model of classification and regression analysis in random forest was applied for mapping soil depth functions using environmental covariates across the study area. Third, SOC stocks were predicted by using soil depth functions at each location.

Generally, the SOC content decreased with depth. The exponential decay function was the most popular depth model for fitting SOC depth distribution^8^,^21^,^23^. A monotonic decreasing function may result in an unrealistic distribution of SOC in the mattic epipedon because of high SOC content in this layer. Alternatively, the SOC content was assumed to be constant until the mattic depth is reached (Eq. 3). Then, the SOC content at deeper depth was fitted with an exponential decay function (Eq. 3):


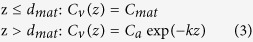


where 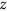 is the absolute depth from the soil surface (m), *d*_*mat*_ is the depth of the mattic epipedon (m), *C*_*mat*_ is the SOC content (kg m^−3^) in the mattic epipedon, *C*_*v*_(*z*) is the fitted SOC content (kg m^−3^) in depth *z*, *k* (>0) controls SOC decrease rate with depth, and *C*_*a*_ is the SOC content at the soil surface (*C*_*a*_ = *C*_*mat*_ in the soils with the mattic epipedon). Thus, the defined soil depth function contains four parameters: occurrence of the mattic epipedon (categorical variable), mattic depth, *C*_*a*_ and *k*.

To model the relationships between the parameters of the soil depth function and environmental covariates, classification and regression techniques in random forest (RF) were used. The RF model is based on classification and regression trees (CART) method to improve prediction accuracy^47^. In model building process, RF grows a numerous trees (ntree) to guarantee model stability, which benefits from bootstrap sampling technique used in RF. Bootstrap sampling results in a random subset of the original training data for building each tree. Only a randomly chosen subset of predictors (mtry) is used to produce the best split. For model building, the number of randomly selected environmental covariates was set as four for classification (mtry = 4) and six for regression (mtry = 6). The number of trees was 1000 in this case. The prediction for regressions is the average of all tree results, while it is the majority of the correct classified outputs for classifications. In addition, the RF algorithm estimates predictor importance by measuring the mean decrease in prediction accuracy.

The spatial distribution of SOC stocks in the upper 1 m depth was mapped using the predicted structure of soil depth functions with a 90-m resolution grid. To estimate total SOC stocks for entire study area, the map of SOC stocks was multiplied by the pixel area, and then SOC stocks at all sites were summed.

For prediction model evaluation, error estimates in RF are achieved by using the out-of-bag (OOB) sample that is not included in the bootstrap sample. The mean square error (MSE) and error rate were estimated by aggregating the OOB predictions for regressions and classifications, respectively. The OOB measurement for prediction accuracy is comparable to k-fold cross-validation^48^,^49^.

In addition, the prediction of SOC stocks was validated with point data, which consists of 96 calibration points and 3 intensively sampled (5-cm) points as independent validation set. Three indices were calculated: the mean error (ME), indicating the biased prediction; the root mean square error (RMSE), measuring the overall quality of the prediction and Lin’s concordance correlation coefficient (LCCC) measuring the degree of predicted and observed values follow the 45° line^50^:


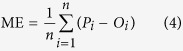



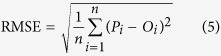



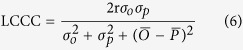


where 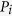 and *O*_*i*_ are the predicted and observed SOC stocks; 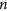 is the number of samples; 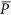 and 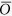 are the means for the predicted and observed SOC stocks; 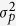 and 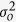 are the variances of predicted and observed values and 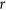 is the Pearson correlation coefficient between the predicted and observed values.

### Software

Raster maps of environmental covariates were processed using ArcGIS 10.0 (ESRI Inc., USA) and SAGA GIS software^51^. Modelling part of this research was performed in R software^52^ using the “randomForest” package^53^.

## Additional Information

**How to cite this article**: Yang, R.-M. *et al.* Precise estimation of soil organic carbon stocks in the northeast Tibetan Plateau. *Sci. Rep.* **6**, 21842; doi: 10.1038/srep21842 (2016).

## Figures and Tables

**Table 1:** Summary statistics of soil organic carbon contents and stocks at surface layers.

	Depth (cm)		SOC content (kg m^#x02212;3^)		SOC stock (kg m^−2^)	
	Mean	SD	Mean	SD	Mean	SD
Mattic epipedon	14.97	4.15	38.06	12.04	5.90	3.24
Ordinary horizon	12.34	5.84	22.15	15.68	2.91	2.48

**Table 2:** Error estimates from random forest models.

Parameters	Indices	Minimum	1st quartile	Mean	Median	3rd quartile	Maximum	SD
Mattic horizon	Error rate	0.15	0.16	0.17	0.17	0.18	0.19	0.008
Log(mattic depth, m)	MSE	0.03	0.04	0.04	0.04	0.04	0.04	0.000
Log(C_a_, kg m^−3^)	MSE	0.34	0.35	0.35	0.35	0.35	0.36	0.002
Log(k)	MSE	0.65	0.66	0.67	0.67	0.67	0.68	0.004

Error rates of the out-of-bag sample for mattic horizon classification and out-of-bag mean square error (MSE) for the predictions of mattic depth and the parameters of exponential depth functions from 100 runs.

**Table 3:** Prediction accuracy.

	Indices	Minimum	1st quartile	Mean	Median	3rd quartile	Maximum	SD
Training	ME	−0.07	−0.06	−0.06	−0.06	−0.05	−0.04	0.005
	RMSE	1.33	1.34	1.35	1.35	1.35	1.36	0.006
	LCCC	0.89	0.89	0.90	0.90	0.90	0.90	0.001
Independent	ME	−0.60	−0.59	−0.59	−0.59	−0.58	−0.57	0.004
	RMSE	0.92	0.93	0.94	0.94	0.94	0.95	0.004
	LCCC	0.53	0.53	0.54	0.54	0.54	0.55	0.004

Mean error (ME), root mean square error (RMSE) and Lin’s concordance correlation coefficient (LCCC) of soil organic carbon stocks (kg m^−2^) evaluated on training and independent dataset.

**Table 4:** Summary of soil organic carbon stocks at the upper 1 m depth in the northeast of the Tibetan Plateau.

Layer	Average SOC (kg m^−2^)	Total SOC stock (Tg)	Relative SOC stock (%)
Mattic horizon	4.99	43.95	20.94
0–30 cm	5.54	168.89	80.48
0–50 cm	6.11	186.22	88.73
0–100 cm	6.89	209.87	100
